# Low incidence of positive smooth muscle antibody and high incidence of isolated IgM elevation in Chinese patients with autoimmune hepatitis and primary biliary cirrhosis overlap syndrome: a retrospective study

**DOI:** 10.1186/1471-230X-12-1

**Published:** 2012-01-03

**Authors:** Pan Zhao, Yukun Han

**Affiliations:** 1 Liver Failure Therapy and Research Center, Beijing 302 Hospital, Beijing 100039, China; 2 Case-history Office, Beijing 302 Hospital, Beijing 100039, China

**Keywords:** autoantibody, overlap syndrome, autoimmune hepatitis, primary biliary cirrhosis

## Abstract

**Background**

Up to now, few data are available regarding the clinical characteristics of autoimmune hepatitis and primary biliary cirrhosis overlap syndrome. The study was to investigate and analyze the prevalent and clinical features of Chinese patients with this disease.

**Methods**

Clinical data on patients diagnosed as autoimmune hepatitis and primary biliary cirrhosis overlap syndrome in our hospital from January 2001 to December 2006 were collected and analyzed.

**Results**

Overlap syndrome of autoimmune hepatitis and primary biliary cirrhosis accounted for 10.33% of patients with autoimmune liver diseases during the past six years. For these patients with overlap syndrome, xanthochromia, lethargy and anorexia were the predominant complaints; a low incidence (14/146) of smooth muscle antibody positivity and a high incidence (37/89) of isolated IgM elevation were the main serological characteristics.

**Conclusions**

Overlap syndrome of autoimmune hepatitis and primary biliary cirrhosis was not rare in Chinese patients with clinical manifests of autoimmune liver diseases. Overlap of the diseases should not be disregarded when isolated IgM elevation was exhibited, and smooth muscle antibody might have little diagnostic significance in the overlap syndrome. If it was difficult to make a definite diagnosis, liver biopsy was necessary.

## 1. Background

Autoimmune hepatitis (AIH), primary biliary cirrhosis (PBC), and primary sclerosing cholangitis (PSC) are three major clinicopathologic entities of autoimmune liver diseases. Besides, some patients with autoimmune liver disease present with characteristics of a second autoimmune liver disease (i.e. AIH and PBC overlapping). These cases have been defined as overlap syndrome. Patients with overlap syndrome usually present with nonspecific symptoms, including lethargy, arthralgias, and myalgias. A combination of clinical and laboratory or pathologic criteria is necessary for the diagnosis of the disease. In the laboratory test, autoantibodies are the serological hallmarks. Serum antinuclear antibody (ANA), smooth muscle antibody (SMA) and antimitochondrial antibody (AMA) are routinely detected in these patients [1].

Although combined features of both PBC and PSC have been reported in single cases, there is no clear evidence for the existence of an overlap of PBC and PSC [2]. The overlap syndrome of AIH and PBC is the most common form, and it shows a more progressive course towards liver cirrhosis and liver failure than AIH or PBC alone [3-5]. However, up to now, the pathogenesis of overlap syndrome is poorly understood [6], and few data are available regarding the clinical characteristics of this disease, and moreover, reports on the prevalent and serological features of this condition in Chinese population are still lacking. The aim of this retrospective study is to investigate and analyze the prevalent and clinical features of Chinese patients with AIH and PBC overlap syndrome.

## 2. Methods

### 2.1. Patients

Patients diagnosed as overlap syndrome of AIH and PBC in 302 hospital from January 2001 to December 2006 were included in the retrospective study. The diagnosis was based on the criteria established by Chazouillères O, et al [7]. Exclusion criteria included coinfection with hepatitis A, C, D, E, Epstein-Barr virus, cytomegalovirus or HIV; the presence of other forms of liver diseases such as alcoholic liver disease, drug hepatitis or Wilson's disease. 146 patients were eventually enrolled in our study. Informed written consent for the analysis was obtained from each patient. The study was approved by the ethics committee of Beijing 302 Hospital.

### 2.2. Serological markers and liver histopathology

Serum autoantibodies, including antinuclear antibody (ANA), smooth muscle antibody (SMA) and antimitochondrial antibody (AMA) were tested using indirect immunofluorescence with the standard methods (Euroimmun Medizinnische Labordiagnostika AG, Germany), and sera were considered to be positive when they produced a reaction at a dilution of ≥ 1:100. Immunoglobulin (Ig) assay were taken with the mothod of immunological turbidimetry (Diasys Diagnostic Systems, China). The normalized levels of IgG, IgM and IgA were respectively 7.23-16.6 g/L, 0.63-2.77 g/L and 0.69-3.82 g/L.

Biochemical profiles, including alanine transarninase (ALT), aspartate aminotransferase (AST), total bilirubin (TBil), gamma glutamyl transferase (GGT) and alkaline phosphatase (ALP) were measured using standard laboratory procedure. The normalized levels of ALT, AST, TBil, GGT and ALP were respectively < 40 U/L, < 40 U/L, < 17.1 umol/L, 7-32 U/L, and 40-150 U/L.

Liver biopsy was performed in some cases for definite diagnosis, and biopsy specimens were examined in the Pathology Department.

### 2.3. Statistical analysis

Data analysis were performed using SAS 9.2 software (SAS Institute Inc., Cary, NC, USA) and the quantitative data were expressed as mean and standard deviation.

## 3. Results

### 3.1. Baseline characteristics and prevalent features

In this six-year retrospective survey based on our hospital, a total of 1413 patients was diagnosed as autoimmune liver diseases, of which, 577 were AIH, 685 were PBC, 5 were PSC, 146 were overlap syndrome of AIH and PBC, and none were overlap syndrome of AIH and PSC. Overlap syndrome of AIH and PBC accounts for 10.33% of patients with autoimmune liver diseases, and this proportion was similar to that in previous reports in India and France [8,9]. The characteristics of the 146 patients at the diagnosis of overlap syndrome of AIH and PBC were described in Table 1.

**Table 1:** Characteristics of patients at the diagnosis of AIH and PBC overlap syndrome

	Value
sex: female/male	125/21
age (years)	46.50 ± 10.08
TBil (umol/L)	43.08 ± 27.94
ALT (U/L)	116.80 ± 37.10
AST (U/L)	77.02 ± 32.75
GGT (U/L)	369.12 ± 329.32
ALP (U/L)	399.40 ± 221.53
IgM	3.91 ± 2.08
IgG	18.79 ± 1.56
	n = 146

ALT, alanine transarninase; AST, aspartate aminotransferase; TBil, total bilirubin; GGT, gamma glutamyl transferase; ALP, alkaline phosphatase; Ig, immunoglobulin.

### 3.2. Clinical features

We viewed the 146 case histories, and found that, as the chief complaints at the diagnosis, xanthochromia occurs in 86 patients, with the incidence of 58.90%; lethargy occurs in 79 patients, with the incidence of 54.11%; anorexia occurs in 75 patients, with the incidence of 51.37%; pruritus occurs in 51 patients, with the incidence of 34.93%; discomfort in hepatic region occurs in 33 patients, with the incidence of 22.60%; arthralgias or myalgias occurs in 33 patients, with the incidence of 22.60%; asymptomatic elevation of serum liver enzymes occurs in 20 patients, with the incidence of 13.70%; splenomegaly occurs in 12 patients, with the incidence of 8.22%. Details were summarized in Table 2.

**Table 2:** Chief complaints of patients at the diagnosis of AIH and PBC overlap syndrome

Chief complaint	Case	Incidence (%)
Xanthochromia	86	58.90
Lethargy	79	54.11
Anorexia	75	51.37
Pruritus	51	34.93
discomfort in hepatic region	33	22.60
arthralgias or myalgiasg	33	22.60
asymptomatic elevation of serum liver enzymes	20	13.70
splenomegaly	12	8.22
	n = 146	

Of the 146 patients, 140 had positive ANA, accounting for 95.89%; 129 had positive AMA, accounting for 88.36%; 14 had positive SMA, accounting for 9.59%. Details were seen in Table 3.

**Table 3:** Features of autoantibodies in patients

Features	**No**.	Incidence (%)
ANA positive	140	95.89
AMA positive	129	88.36
SMA positive	14	9.59
both ANA and AMA positive	122	83.56
both SMA and AMA positive	10	6.85
	n = 146	

ANA, antinuclear antibody; AMA, antimitochondrial antibody; SMA, smooth muscle antibody.

A total of 89 patients with immunoglobulin assay was identified. Of these patients, 37 had isolated IgM elevation, accounting for 41.57%; 17 had isolated IgG elevation, accounting for 19.10%; 23 had both IgM and IgG elevation, accounting for 25.84%; 12 had neither IgM nor IgG elevation, accounting for 13.48%. The results were summarized in Table 4.

**Table 4:** Features of immunoglobulin (Ig) elevation in patients

Features	**No**.	Percentage (%)
isolated IgM elevation	37	41.57
isolated IgG elevation	17	19.10
both IgM and IgG elevation	23	25.84
neither IgM nor IgG elevation	12	13.48
	
Total	89	100.00

**Figure 1:**
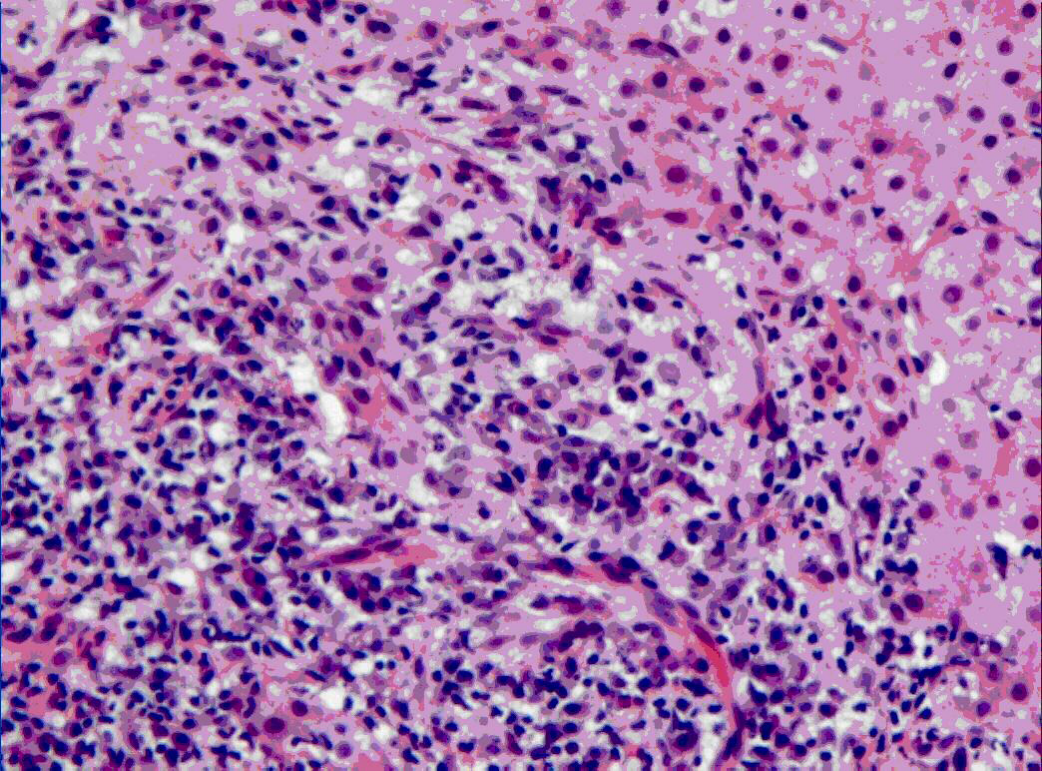
**Typical pathological changes (bile duct lesion, interface hepatitis and plasma cell infiltration) in a Chinese patient diagnosed as AIH and PBC overlap syndrome**. (HE, ×200).

58 patients underwent liver biopsy. Diagnostic pathological changes, including bile duct lesion, interface hepatitis and plasma cell infiltration were observed in all patients. Summarization of the pathological features for these patients was seen in Yanling Sun's previous study [10]. Pathological changes in a patient with AIH and PBC overlap syndrome were shown in Figure 1.

## 4. Discussion

The worldwide prevalence of overlap syndrome of AIH and PBC is unknown. The first cases of this disease were reported almost 30 years ago, and this entity was assumed to be rare [11]. However, in our hospital, 10.33% of patients with autoimmune liver diseases were diagnosed as overlap syndrome of AIH and PBC based on the clinical manifests, laboratory test, and liver biopsy during the six years. Our study also showed that, similar to other studies [12,13], overlap syndrome of AIH and PBC was more common in female patients, with a female:male ratio of roughly 6:1. However, the median age of patients with overlap syndrome was older than that of a previous report about European patients [9].

Presentation of autoimmune liver diseases varies widely, ranging from asymptomatic elevations of serum liver enzymes to massive hepatic necrosis resulting in fulminant hepatic failure, and there are no disease-specific clinical features. Günsar F, et al [14] reported that, lethargy was the most common symptom in these patients. Our study showed that xanthochromia, lethargy and anorexia were the predominant three chief complaints, that was, besides lethargy, xanthochromia was also a most common symptom in Chinese patients with overlap syndrome of AIH and PBC.

Serum autoantibodies have steadily established themselves as critical biomarkers for the diagnosis of autoimmune diseases [15]. For the North-American and European population, ANA and SMA constitute the standard repertoire for the diagnosis of autoimmune hepatitis, and AMA is the diagnostic marker of primary biliary cirrhosis [7,16]. In our study, the majority of patients had positive serum ANA (140/146) and AMA (129/146), whereas only minor patients (14/146) had positive serum SMA. This result showed that, there was a low incidence of positive serum SMA in Chinese patients with overlap syndrome of AIH and PBC, which might indicate that serum SMA may have little diagnostic significance in the overlap syndrome for Chinese patients. This finding agreed with another investigation performed by Zhenxia Liu [17].

Some studies had shown that the serum immunoglobulins could elevate in most cases of autoimmune liver diseases [18-22] and types of the elevated immunoglobulins were distinctive in different categories of autoimmune liver diseases. IgG was the predominant immunoglobulin elevated in serum of AIH patients while IgM was elevated in most patients with PBC [23-25]. However, in our study, isolated IgM elevation was preponderant (37/89), which presented with features of PBC. Previously, a German study showed that patients with overlap syndrome of AIH and PBC presented with typical features of PBC when compared to AIH and PBC patients [13]. The reason, we inferred, might be that serological markers were often featured with one predominant pathological change to the other in overlap syndrome. But how to distinguish the predominant one needed further studies.

## 5. Conclusion

Overlap syndrome of autoimmune hepatitis and primary biliary cirrhosis was not rare in Chinese patients with clinical manifests of autoimmune liver diseases. Overlap of the diseases should not be disregarded when isolated IgM elevation was exhibited, and smooth muscle antibody might have little diagnostic significance in the overlap syndrome. If it was difficult to make a definite diagnosis, liver biopsy was necessary.

## List of abbreviations

AIH: autoimmune hepatitis; ALP: alkaline phosphatase; ALT: alanine transarninase; AMA: antimitochondrial antibody; ANA: antinuclear antibody; AST: aspartate aminotransferase; GGT: gamma glutamyl transferase; PBC: primary biliary cirrhosis; PSC: primary sclerosing cholangitis; SMA: antismooth muscle antibody; Tbil: total bilirubin.

## Competing interests

The authors declare that they have no competing interests.

## Authors' contributions

YH designed the study; PZ analyzed the data and wrote the manuscript. PZ and YH were both involved in the acquisition of the data. All authors read and approved the final manuscript.

## Pre-publication history

The pre-publication history for this paper can be accessed here:

http://www.biomedcentral.com/1471-230X/12/1/prepub
